# Association pernicious anemia and autoimmune polyendocrinopathy: a retrospective study

**Published:** 2017

**Authors:** AA Zulfiqar, E Andres

**Affiliations:** * Department of Internal Medicine, Geriatrics, Therapeutics, CHU Rouen, France; ** Département de Médecine Interne, CHRU Strasbourg, France

**Keywords:** autoimmune polyglandular syndrome, autoimmune thyroiditis, pernicious anemia

## Abstract

Objective: To investigate the association between pernicious anemia and other autoimmune diseases.

Methods: This retrospective and bicentric study was conducted at Reims and Strasbourg University Hospitals and involved 188 patients with pernicious anemia examined between 2000 and 2010 in order to search for other autoimmune diseases and to evaluate the role of pernicious anemia in autoimmune polyglandular syndrome.

Results: A total of 74 patients with a combination of pernicious anemia and other autoimmune diseases were included in the study. Our study revealed the privileged association of pernicious anemia with autoimmune thyroiditis. The association of pernicious anemia and autoimmune thyroiditis are a part of the autoimmune polyglandular syndrome type 3b.

Conclusion: We suggest undertaking a systematic clinical examination and laboratory investigations in search of autoimmune thyroiditis in patient(s) with the diagnosis of pernicious anemia. The association of pernicious anemia and autoimmune thyroiditis is frequent and a part of autoimmune polyglandular 3b.

## Introduction

Patients with pernicious anemia or their 1st degree relatives very often suffer from a number of autoimmune diseases. They may precede or occur with pernicious anemia at the waning of the disease.

The association with other autoimmune diseases such as type 1 diabetes, autoimmune thyroiditis, or vitiligo is quite common [**1**]. The main goal of this study was to investigate the association of pernicious anemia and other autoimmune diseases and the prevalence of autoimmune diseases associated with pernicious anemia.

## Methods

This was a retrospective, descriptive, multicentered, observational study of 188 patients suffering from pernicious anemia, diagnosed and/or dealt with in several departments of Medicine of the University Hospital of Reims (Internal Medicine - Infectious Diseases - Medical Gastroenterology - Endocrinology) and in the department of Internal Medicine of the University Hospital of Strasbourg between January 2000 and December 2010. The records of 188 patients were studied retrospectively in search of autoimmune diseases associated with pernicious anemia. Clinical research for other illnesses has been systematically carried out by an examination, a complete physical examination, and appropriate investigations, at the time of diagnosis of pernicious anemia, or when there was a clinical suspicion.

**Criteria for inclusion**

All the patients included in this retrospective study suffered from symptomatic pernicious anemia, with the two following criteria being applied for inclusion:

- Diagnosis of fundal atrophic gastritis: reduced levels of mucus in the fundus together with elongated crypts and rarefaction of the glands, which are small in size, with varying amounts of inflammatory infiltration of the periglandular lymph nodes, observed intestinal metaplasia, linear or micronodular hyperplasia of the endocrine cells, made evident as a result of Grimelius staining or by anti-chromogranin A immunolabeling; all in the absence of an infection of Helicobacter pylori.

- Criteria for autoimmune syndromes: presence of antibodies against the parietal cells (with positivity being determined at a level ≥ 1/100) and/or anti-intrinsic factor antibodies (positivity for rates > 20 µI/ml).

**Exclusion criteria**

Patients not meeting the two criteria for inclusion set out above were excluded.

**The records of 188 patients were retrospectively investigated for autoimmune conditions associated with pernicious anemia.**

Clinical research into other associated pathologies was performed systematically by way of interview, a full clinical examination and appropriate complementary examinations at the time that pernicious anemia was diagnosed, or independently, in advance or retrospectively upon the identification of possible clinical indications.

The diagnosis of an autoimmune disease was based on the 1997 criteria published by the American College of Rheumatology for lupus [**2**], while the diagnosis of Sjögren syndrome (SjS) was based on the criteria published by Vitali (2002) [**3**].

Diagnosis of autoimmune thyropathy was based on clinical examination and the presence of autoimmune anti-thyroid antibodies (anti-thyroglobulin (TG) and/or anti-thyroid peroxidase (TPO) antibodies). Dosage methods used were microparticle immunofluorescence, coupled with flow cytometry, while a radio-immunological method was used for the dosing of anti-TSH receptor antibodies.

Type 1 diabetes was diagnosed in the presence of a diabetic condition requiring insulin treatment in response to recent indications of hypoinsulinism with the presence of autoimmune stigmata and characterized by the presence of islet-cell antibodies (ICA) - dosage method: IFI on the human pancreas or anti-glutamic acid decarboxylase (GADA) - dosage method: ELISA GAD IgG EUROIMMUN.

Diagnosis of autoimmune hemolytic anemia (AIHA) was based on the presence of anemia in combination with criteria of elevated intravascular hemolysis and positive results from a direct Coombs test.

Diagnosis of Addison’s disease was based on clinical data, a hormonal analysis, and the presence of anti-adrenal antibodies.

Diagnosis of vitiligo was based on clinical interview and examination of the patient.

Diagnosis of antiphospholipid syndrome (APLS) was based on international criteria (2006) [**4**].

Diagnosis of myasthenia was based on data generated by interview, clinical examination, electromyography, and on the detection of anti-acetylcholine receptors.

Diagnosis of autoimmune hepatitis (AIH) was based on clinical data, the presence of biological cholestasis, hepatic histology and the presence of non-specific autoimmune antibodies detected by IFI on a triple substrate (anti-mitochondria antibodies, anti-liver kidney microsomal (LKM) antibodies, anti-hepatic cytosol antibodies or anti-smooth muscle antibodies).

**Processing of data**

Centralized data entry was undertaken using a Microsoft Excel spreadsheet. Control was performed for anomalous or missing data.

Statistical analysis was undertaken using SAS for Windows version 9.2 software (SAS Institute).

The threshold for statistical significance was set at p<0.05.

## Results

Out of the 188 patients with pernicious anemia, 74 patients with a combination of pernicious anemia and other autoimmune diseases were included in the study. The average age was 61 years old (e-t = 17.3, range = 25-98), 61 subjects (82.4%) were women; the sex ratio (Male/Female) was 0.2. Our study counted 74 cases of pernicious diseases associated with one or more autoimmune diseases. Our series revealed the privileged association of pernicious anemia with autoimmune thyroiditis in 57 of 74 cases (12 Graves and 45 Hashimoto), with a female predominance. The concomitant diagnoses pernicious anemia/autoimmune thyroiditis has been recorded for 23 cases. We described our series in **Table 1**.

**Table 1:** Frequency of autoimmune diseases in patients with pernicious anemia (n=74)

AID	n	%	Male(n)	%	Female (n)	%	p
Graves	12	16.2	1	7.7	11	18	0.36
Hashimoto	45	60.8	9	69.2	36	59	0.5
AID	23	31.1	5	38.5	18	29.5	0.5
Vitiligo	9	12.2	0	0	9	14.75	0.14
GS	7	9.5	0	0	7	11.5	0.2
APLS	5	6.8	1	7.7	4	6.6	0.9
AIH	1	1.35	0	0	1	1.6	0.6
Addison	5	6.8	0	0	5	8.2	0.3
Myasthenia	2	2.7	0	0	2	3.3	0.5
SLE	1	1.35	0	0	1	1.6	0.64
AIHA	2	2.7	1	7.7	1	1.6	0.2
p = male/female AID = autoimmune diseases; AID = autoimmune diabetes; GS = Gougerot-Sjogren; APLS = antiphospholipid syndrome; AIH = autoimmune hepatitis; SLE = systemic lupus erythematosus; AIHA = autoimmune hemolytic anemia							

## Discussions

The association between pernicious anemia and autoimmune thyroiditis was frequent in our study. It was observed in a series of 78 patients with pernicious anemia. Markson et al. have noted positive anti-thyroid antibodies in 33% of the cases [**5**] and Doniach et al. in a series of 100 patients with positivity in 47% of the cases [**6**]. There is certainly a pathogenetic link between pernicious anemia and autoimmune thyroiditis. In our study, the diagnosis of pernicious anemia and autoimmune thyroiditis was concomitant in 23 cases. However, pernicious anemia is sometimes preceded by the autoimmune thyroiditis by several years. Based on the results of our study, we believe an evaluation of thyroid function should be performed routinely in a patient suffering from pernicious anemia. Other studies also cite the improvement of early detection in both patients with pernicious anemia and autoimmune thyroiditis. A study by Nicolino-Peltier et al. on the detection of pernicious anemia in a group of 120 patients with autoimmune thyroid disease helped the probable diagnosis of pernicious anemia in pre-stage anemia in 8 patients of the 120 patients with autoimmune thyroiditis disease (including a balance sheet looking for anti-gastric mucosal antibodies, assay of gastrin and vitamin B12 by radioimmunoassay) [**7**]. Morel et al. [**8**] studied the prevalence of the autoimmune thyroid disease and pernicious anemia combination according to the presence of anti-intrinsic factor antibodies in patients suffering from autoimmune thyroiditis. They found a prevalence of anti-intrinsic factor antibodies at 3.5%, being higher in patients with autoimmune thyroid disease than in those with non-autoimmune thyroid dysfunction [**9**]. Our study found a pernicious anemia/autoimmune diabetes combination in 23 (31%) of the cases.

There were 15 cases among these 23 associations, in which an autoimmune thyroiditis was associated with it. Autoimmune diabetes is often associated with autoimmune thyroiditis; the prevalence of autoimmune thyroiditis varies from 15 to 30% of autoimmune diabetes subjects [**10**]. An estimated prevalence of pernicious anemia in patients with autoimmune diabetes is in the range of 5 to 10% [**10**]. A study by Perros et al. [**1**] of 63 patients with autoimmune diabetes associated with autoimmune thyroiditis aimed to determine the prevalence of pernicious anemia in these patients. The prevalence was estimated at 6.3%, while the prevalence rises to 8.5% in women [**1**]. This helped us conclude that patients with both autoimmune diabetes and autoimmune thyroiditis are at risk to develop pernicious anemia, which was confirmed in our study. Vitiligo was present in 9 subjects with pernicious anemia (12%). Vitiligo appears to be the most common skin disease [**10**], and this is what appeared in our study. Apart from pernicious anemia, vitiligo appears to be preferentially associated with autoimmune thyroiditis, association evidenced by the study of Klisnick et al. [**10**].

Therefore, we found a preferential association of pernicious anemia and autoimmune thyroiditis, with a female predominance and concomitant in 23 cases in our series. Pernicious anemia can be part of a large group of autoimmune polyendocrinopathy characterized by the coexistence of two or more endocrine deficiencies related to an autoimmune mechanism, sometimes associated with non-endocrine disease [**11**]. Type 1 polyendocrinopathy is rare, affecting small children, and is characterized by the coexistence of chronic candidiasis, hypoparathyroidism, and acquired peripheral adrenal insufficiency. This is a monogenic autosomal recessive syndrome determined by mutations in the autoimmune regulator gene AIRE (autoimmune regulator), recently identified on chromosome 21q22.3 [**12**]. The AIRE gene is expressed in the thymus, lymph nodes, leukocytes, pancreas, and adrenal cortex. It is a nuclear transcription factor whose mutations are the cause of a disruption of immune tolerance. Autoimmune polyglandular type 2 is characterized by a combination of primary adrenal insufficiency with thyroid disease carrying the Schmidt syndrome and more or less type 1 diabetes carrying the Carpenter syndrome in adults [**11**]. The main syndrome characterizes autoimmune polyglandular type 3, which is autoimmune thyroiditis. It can join either autoimmune diabetes (and more or less sarcoidosis or celiac disease), thus describing the autoimmune polyglandular syndrome type 3a, either pernicious anemia defining type 3b or vitiligo and alopecia, which define type 3c. It differs from type 2 by the absence of adrenal insufficiency. The studied association of pernicious anemia/autoimmune thyroiditis is part of the autoimmune polyglandular syndrome type 3b [**13**]. Both cases represent polygenic syndromes with autosomal dominant transmission and incomplete penetrance. The presence of histocompatibility antigen HLA-DR3 is increased, especially the DQB1.0102-DR3 subtype [**14**]. In our study, pernicious anemia was well integrated as follows. We noted five cases in which pernicious anemia was associated with adrenal insufficiency, autoimmune thyroiditis and autoimmune diabetes (inconsistently), defining type 2. What emerged from our study was that pernicious anemia fits more frequently in the autoimmune polyglandular syndrome type 3 with 30 pernicious anemia/autoimmune thyroiditis associations (defining type 3b), 8 cases of pernicious anemia-autoimmune thyroiditis-autoimmune diabetes, 2 cases of pernicious anemia-autoimmune thyroiditis-myasthenia, 3 cases pernicious anemia-autoimmune thyroiditis-autoimmune diabetes-vitiligo, one case of pernicious anemia-autoimmune thyroiditis-vitiligo, 2 cases pernicious anemia-autoimmune thyroiditis-Gougerot-Sjogren and 1 case of pernicious anemia-autoimmune thyroiditis-antiphospholipid syndrome. Autoimmune thyroiditis is the cornerstone of type 3 autoimmune polyglandular syndrome.

The HLA locus is the main example of participation in a common genetic background with a large number of autoimmune diseases. HLA molecules are encoded by the major histocompatibility complex on chromosome 6 and are essential in the immune system, playing a central role in antigen presentation to T cells. For most autoimmune diseases, HLA is to date the genetic factor with the greatest weight in the genetic component with a relative risk ranging from 8 to 20 [**15**][**16**]. Thus, HLA associations and disease is the link that unites the various autoimmune diseases [**14**]. Pernicious anemia has an association with HLA-DR5 for a relative risk of 5, Hashimoto's thyroiditis partnering with the HLA-DR3 for a relative risk of 3.2 and HLA-DR5 for a relative risk of 5.8 [**17**], the autoimmune diabetic subjects, 90% of the Caucasian population associated with HLA-DR3 and/or DR4. Haplotypes risks of Hashimoto's thyroiditis are HLA DQ A1 * 0301 linked to DR4, DQB1 * 0301 associated with DR5 and DQB1 * 0201 linked to DR3 [**9**]. The haplotype DR3- DQB1 * 0201 contributes to genetic susceptibility of type 1 diabetes, autoimmune thyropathy and polyglandular 2 and 3 [**9**]. A genetic predisposition has been suggested for pernicious anemia. A weak association between pernicious anemia and HLA haplotype DQA1 * 0501 -B1 * 0301, HLA-DR5 bound was observed in type 1 diabetic subjects. Patients who associate pernicious anemia and autoimmune endocrine diseases often have the DR3/DR4 genotype. The association of pernicious anemia and autoimmune thyroiditis is part of a typical type 3 autoimmune polyglandular syndrome for which a genetic predisposition (HLA- B8 and/or DR3 and DR5) seems important.

These data support the involvement of the DR3 haplotype in the susceptibility to autoimmune diseases. HLA DR3 could be a thread in the immunogenetic association of various autoimmune diseases in the autoimmune polyglandular syndrome.

The association between pernicious anemia and autoimmune thyroiditis suggests that there should be a common pathogenetic mechanism indicating an autoimmune phenomenon including the thyroid and stomach, probably due to an altered immune response in genetically predisposed individuals [**18**].

## Conclusions

The association of pernicious anemia and autoimmune thyroiditis is not fortuitous as evidenced by the large number of cases observed in our series and the literature data. We suggest undertaking a systematic clinical and laboratory investigation in search of an autoimmune thyropathy in patient(s) with the diagnosis of pernicious anemia and vice versa. We believe that evaluation of the thyroid function should be performed routinely in a subject in whom pernicious anemia is discovered. Similarly, a patient with autoimmune thyroid disease should be regularly monitored for several years to detect an autoimmune disease such as pernicious anemia as soon as possible. The HLA-DR3 test should be systematically performed in patients suffering from pernicious anemia or autoimmune thyropathy, opening a door for screening.

## Conflict of interest

The authors declare no conflict of interest.
